# Loop-Mediated Isothermal Amplification assays for on-site detection of the main sweetpotato infecting viruses

**DOI:** 10.1016/j.jviromet.2021.114301

**Published:** 2021-12

**Authors:** Bramwel W. Wanjala, Elijah M. Ateka, Douglas W. Miano, Segundo Fuentes, Ana Perez, Jan W. Low, Jan F. Kreuze

**Affiliations:** a International Potato Center, SSA Regional Office, PO Box 25171, 00603, Nairobi, Kenya; b Jomo Kenyatta University of Agriculture and Technology, P.O. Box 62000, 00200, Nairobi, Kenya; c University of Nairobi, P.O. Box: 30197, 00100, Nairobi, Kenya; d International Potato Center, Avenida La Molina 1895, La Molina, Apartado Postal 1558, Lima, Peru

**Keywords:** Detection, LAMP assay, On-site, SPFMV, SPCSV, SPLCV (begomoviruses)

## Abstract

Globally, Sweet potato feathery mottle virus (SPFMV) and Sweet potato chlorotic stunt virus (SPCSV) occur frequently and in combination cause sweetpotato virus disease (SPVD). Many viral diseases are economically important and negatively impact the production and movement of germplasm across regions. Rapid detection of viruses is critical for effective control. Detection and quantification of viruses directly from sweetpotato remains a challenge. Current diagnostic tests are not sensitive enough to reliably detect viruses directly from the plant or require expensive laboratory equipment and expertise to perform. We developed a simple and rapid loop‐mediated isothermal amplification (LAMP) assay for the detection of SPFMV, SPCSV and begomoviruses related to sweet potato leaf curl virus (SPLCV). Laboratory validation recorded 100 % diagnostic sensitivity for all the three viruses. The LAMP assays were customized for field testing using a lyophilized thermostable isothermal master mix in a ready-to-use form that required no cold chain. The average time to positivity (TTP) was: SPFMV 5−30 min, SPCSV 15–43 min s and begomoviruses 28−45 mins. LAMP on-site testing results were comparable to PCR and RT-PCR confirmatory laboratory tests. The LAMP assay is a powerful tool for rapid sweetpotato virus detection at a reasonable cost and thus could serve as quality control systems for planting materials.

## Highlights

• LAMP assays were developed to detect the three main sweetpotato viruses.
• Unpurified extracts could be used, and results were obtained from 5 to 45 minutes.
• The sensitivity was higher than RT-PCR and PCR assays.
• The assays showed 100 % concordance with standard virus indexing in laboratory.
• On-site, in-field testing at different location in Kenya demonstrated high accuracy.

## Graphical abstract

LAMP on-site sweetpotato virus detection workflow.

**Figure:**
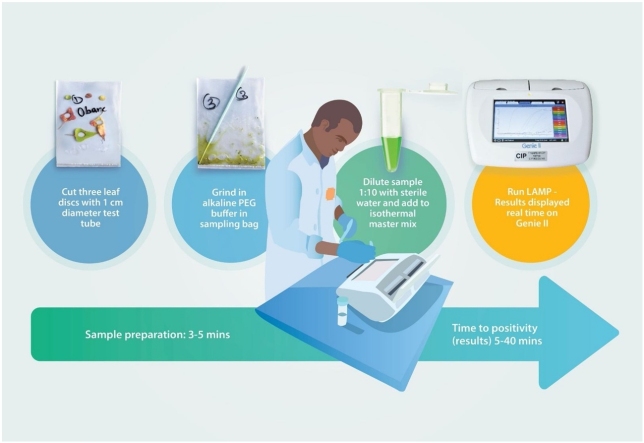

## 1 Introduction

Viral diseases occur the world over and are a major constraint to sweetpotato production. To date, more than 30 viruses have been reported to infect sweetpotato (Clark et al., 2012; Kwak et al., 2014). Sweetpotato virus disease (SPVD) is the most important and most difficult disease to manage (Valverde et al., 2007). SPVD is a result of synergistic interaction between a crinivirus (*Closteroviridae*), Sweet potato chlorotic stunt virus (SPCSV) and a potyvirus (*Potyviridae*), Sweet potato feathery mottle virus (SPFMV). However, several other viruses (mostly potyviruses) are also known to cause synergistic diseases with SPCSV (Kreuze and Fuentes, 2008; Clark et al., 2012). In addition, Sweetpotato leaf curl virus (SPLCV) and related viruses belonging to the family *Geminiviridae*, genus *Begomovirus* are recognized as commonly infecting sweetpotato and can cause significant yield losses despite the lack of obvious foliar symptoms (Zhang and Ling, 2011; Wanjala et al., 2020).

The most common test used to detect sweetpotato viruses is the nitro-cellulose membrane enzyme linked immunosorbent assay (NCM-ELISA) (Salazar and Fuentes, 2000; Aritua et al., 2007) and the double antibody sandwich ELISA (DAS-ELISA). However, due to low titres in sweetpotato, it is recommended that infected material is first grafted on the susceptible indicator host *Ipomoea setosa* as it will increase the virus concentration, displaying easily identifiable symptoms and the ELISA will not be affected by inhibitors found in sweetpotato sap (Kreuze and Fuentes, 2008). However, it requires skilled personnel at the stages of grafting and symptom observation and a lot of screen house space. It is also time consuming, taking between 3–6 months to complete the assessment. An NCM-ELISA kit is available from the International Potato Centre in Peru; which tests for ten of the most important sweetpotato viruses (C-6, CMV, SPCaLV (now called SPCV), SPCFV, SPCSV, SPFMV, SPLV, SPMMV, SPMSV and SPVG) (Fuentes et al., 2019a,b). The kit format requires minimal laboratory equipment, can process up to 96 samples and is able to identify the viruses present in a plant. However, there are no antibodies for all the reported viruses (e.g. SPLCV) and the test is only fully reliable when combined with indexing to indicator plants (Dennien, 2015).

Challenges in the detection of sweetpotato viruses have been reported to be caused by low viral titers (Karyeija et al., 2000; Barkley et al., 2011) the occurrence of mixed infections, diverse viral strains (Kreuze and Fuentes, 2008) and how the virus is distributed within the plant. Valverde et al. (2007) further demonstrated that the common presence of SPFMV often masked the presence of other viruses in sweetpotato, especially potyviruses, and hindered efforts to isolate and identify them. However, progress has been made in developing sensitive detection techniques for sweetpotato viruses (Barkley et al., 2011). Nucleic acid-based detection methods include: nucleic acid spot hybridization (NASH), Polymerase chain reaction (PCR) and Reverse transcriptase polymerase chain reaction (RT PCR), and more recently quantitative real time PCR (qPCR), Microarrays, and high-throughput sequencing (Boonham et al., 2014). The testing methods mentioned above have revolutionized virus diagnostics as they are more sensitive, rapid or can detect many viruses at the same time. However, they require lab facilities, specialized staff, costly reagents and equipment (Boonham et al., 2014). On the other hand, lateral flow devices (LFDs) and Loop-mediated isothermal amplification (LAMP) have emerged and could be used in a field set up (Boonham et al., 2014). LAMP stands out as it offers the potential of being cheaper, faster, more robust and adequately sensitive, with minimal processing of samples required, compared to existing laboratory-based tests. This makes it suitable for consideration for on-site or in-field detection of plant viruses.

The loop-mediated isothermal amplification (LAMP) method, developed by Notomi et al. (2000) amplifies nucleic acids relying on the DNA strand displacement activity of DNA polymerase. The method uses four different primers that recognize six distinct independent regions of a target sequence. An improvement has been made by the addition of two loop primers, increasing the sensitivity of the reaction tenfold and reducing the reaction time (Li and Ling, 2014). Several studies have documented that LAMP is approximately 10–100-times more sensitive than PCR, comparable to qPCR, significantly faster (45–60 min) and less sensitive to inhibitors than PCR (Nagamine et al., 2002; Li et al., 2007; Le et al., 2010; Bhat et al., 2013). LAMP is versatile and has the potential to be deployed on-site. Its products can be detected by a number of methods: colorimetric - visualized by color indicators (SYBR Green and hydroxyl naphthol blue – HNB) (Wastling et al., 2010); turbidity of magnesium pyrophosphate formed during the reaction (precipitate) (Wastling et al., 2010); display of ladder-like patterns on an agarose gel electrophoresis (Goto et al., 2009; Pankaew et al., 2019) and recently in real-time, based on intercalating fluorescent dyes (Armson et al., 2019).

LAMP is gaining popularity in plant health diagnostics because of its time efficiency and cost effectiveness. The LAMP assay has been successfully used to detect plant pathogens; phytoplasma infecting papaya, potato, coconut, periwinkle, and some insect hosts (Bekele et al., 2011; Tomlinson, 2012; Ravindran et al., 2012). The current study developed a LAMP assay for the detection of the most common and relevant sweetpotato viruses SPFMV (and ubiquitous potyviruses – SPVG, SPVC and SPV2); SPCSV and begomoviruses related to SPLCV, referred to collectively as SPLCV in the remainder of the manuscript. Further, we evaluated the LAMP assay for on-site detection in different geographical regions in Kenya. The field results were confirmed by RT-PCR and PCR assays.

## 2 Materials and methods

### 2.1 Reference/control and investigated virus samples

Twenty reference samples that tested positive for SPFMV, SPCSV and SPLCV alone or combined were used to optimize the LAMP assays and validated with RT-PCR/ PCR. These comprised samples (n = 10) from the International Potato Centre research support unit, Peru (CIP-RSU Lima) and (n = 10) from the Kenya Plant Health Inspectorate Service, Plant Quarantine and Biosecurity Station, Kenya (KEPHIS-PQBS). The virus/es present in each sample are shown in Table 1. Infection with SPFMV, SPCSV and SPLCV had earlier been confirmed by grafting onto *I. setosa* (Fuentes and Müller, 2019) combined with symptom observation and NCM-ELISA test carried out using a kit manufactured by CIP (Fuentes et al., 2019a,b). Antisera for SPLCV is not available and was instead detected by PCR as described below. The procedure of grafting to *I. setosa,* combined with ELISA and PCR is considered the gold standard, to which to compare any other assays. These samples were used for initial LAMP assay optimizations (Sections 2.3 and 2.4). In addition, samples were selected randomly from field samples collected during surveillance in 2016/2017 from sweetpotato growing regions of western, coastal, eastern and central Kenya and established in screen houses at KEPHIS-PQBS Muguga. They were screened for the presence of SPFMV, SPCSV and SPLCV as described above. To develop and optimize the LAMP assay, 50 samples each, found to be positive for SPFMV, SPCSV and SPLCV alone or in combinations (together comprising 25 samples for each individual virus) and 50 found negative for the three viruses were used. LAMP assays developed were further evaluated in the field to test for operational performance in four sweetpotato growing regions in Kenya – Muguga, Kakamega, Kiboko and Mtwapa, obtaining 24 samples from each site. From each leaf tissue assayed in the field by LAMP assay, the same tissue was preserved by desiccation in filter paper and silica gel and shipped to the lab at KEPHIS-PQBS. To ensure reproducibility during confirmation with RT-PCR (SPFMV, SPCSV – RNA viruses) and PCR (SPLCV – DNA viruses), nucleic acids were extracted with the Ambion kit as described below from the same tissue.

**Table 1:** Reference sweetpotato viruses used in this study and their source.

Lab no number/code	Sample code	Viruses present	Source
1	CIP_1_L	SPFMV^ρ^	CIP RSU Lima*
2	CIP_2_L	SPCV	CIP RSU Lima*
3	CIP_3_L	SPVG^ρ^	CIP RSU Lima*
4	CIP_4_L	SPMMV	CIP RSU Lima*
5	CIP_5_L	SPCFV	CIP RSU Lima*
6	CIP_6_L	SPVD	CIP RSU Lima*
7	CIP_7_L	SPV2^ρ^	CIP RSU Lima*
8	CIP_8_L	SPVC^ρ^	CIP RSU Lima*
9	CIP_9_L	SPC6V	CIP RSU Lima*
10	CIP_10_L	Sweepovirus	CIP RSU Lima*
11	CIP_37_K	SPVD	KEPHIS PQBS^‡^
12	CIP_43_K	SPCSV + SPFMV + SPMMV	KEPHIS PQBS^‡^
13	CIP_23_K	SPCSV + SPVG	KEPHIS PQBS^‡^
14	CIP_42_K	SPFMV	KEPHIS PQBS^‡^
15	CIP_73_K	SPFMV	KEPHIS PQBS^‡^
16	CIP_36_K	SPFMV	KEPHIS PQBS^‡^
17	CIP_16_K	SPFMV + SPCSV + SPMMV	KEPHIS PQBS^‡^
18	CIP_10_K	SPVD + Sweepovirus	KEPHIS PQBS^‡^
19	CIP_28_K	SPFMV + Sweepovirus	KEPHIS PQBS^‡^
20	CIP_97_K	Sweepovirus	KEPHIS PQBS^‡^

Key.

*CIP RSU Lima – International Potato, Centre research support unit, Peru.

^‡^KEPHIS PQBS – Kenya Plant Health Inspectorate Service, Plant Quarantine and Biosecurity Station, Kenya.

^ρ^ Panel of potyviruses used to evaluate the specificity of SPFMV primer. SPFMV- *Sweet potato feathery mottle virus*, SPCV – *Sweet potato virus C*, SPVG – *Sweet potato virus G*, SPV2 – *Sweet potato virus 2*.

SPCSV – *Sweet potato chlorotic stunt virus*; SPVD – SPFMV + SPCSV, SPCFV – *Sweet potato chlorotic fleck virus*, SPVC – *Sweet potato virus C*, SPC6V – *Sweet potato C- 6 virus*, SPMMV – *Sweet potato mild mottle* virus, SPLCV – *Sweet potato leaf curl virus* (Sweepoviruses).

### 2.2 Total nucleic acid extraction

Three leaves (third, fifth and seventh leaves from the top) of a test plant were sampled into 4"x6", 150 microns plastic extraction bags. Three one cm sweetpotato discs (one from each sampled leaf) were sampled in duplicate from the same leaf tissue to compare two parallel nucleic acid extraction methods. Total nucleic acid was extracted from one of the duplicates using the Ambion Kit (Thermo Fischer Scientific, Wilmington, DE, USA), as described by the manufacturer. The kit-extracted RNA/DNA purity and concentration were checked using a NanoDrop ND-1000 Spectrophotometer (Thermo Fischer Scientific) followed by a quality check on 2% agarose gel. RNA/DNA concentrations were standardized to 100 ng/μL before use in amplification assays. For LAMP, we also used the alkaline polyethylene glycol (APEG) quick extraction method (Chomczynski and Rymaszewski, 2006; Blaser et al., 2018) with slight modifications. Briefly, the APEG buffer was prepared by combining 60 g PEG 200 (Sigma-Aldrich) with 0.93 mL of 2 M KOH and 39 mL water and pH adjusted to 13.5. Three 1 cm diameter sweetpotato leaf discs were cut using a 1 cm diameter test tube in the plastic extraction bags and mixed with 1 mL APEG buffer. For root sampling, three root disks from the distal end were sliced with Harris Uni-core 3.00 mm and placed in plastic extraction bags and mixed with 1 mL APEG buffer. Samples were macerated in plastic bags using a test tube and the mixture was left to stand for 1 min for particles to sediment. It was not possible to quantify the nucleic acid concentration from crude APEG extracts, instead they were diluted 1:10 in molecular grade water and used directly for the LAMP assay. The APEG extracts used in LAMP assays were not suitable for RT-qPCR/qPCR or RT-PCR/ PCR assays.

### 2.3 Conventional reverse transcription (RT-PCR) and PCR assays for RNA and DNA viruses

RNA viruses (SPCSV and SPFMV) were detected using a SuperScript III One-Step RT-PCR with Platinum Taq Kit (Invitrogen, California, United States), using the primers described by (Kwak et al., 2014). A 20 μl reaction comprising 2 μl RNA, a mixture of equal amounts of 0.5 μM forward and reverse primers and RT-PCR master mix as recommended by the manufacturer was used. Reaction conditions were: cDNA synthesis for 45 min at 52 °C; initial denaturation at 95 °C for 5 min and 40 cycles consisting of 30 s at 95 °C, 1 min at 55 °C, 1 min at 72 °C, and final extension 5 min at 72 °C. Runs were performed using a GeneAmp 9700 PCR (Applied Biosystems, Foster City, CA, USA). PCR products were analyzed by electrophoresis on a 1.5 % agarose gel, 0.5 X TE; run at 100 V for 1.30 h, stained with GelRed and visualized using a UV transilluminator. DNA virus (SPLCV–sweepoviruses) was tested by PCR as described by Li et al. (2004) using Sweepovirus-specific primers SPG1 and SPG2, designed to amplify a 901-bp fragment. In addition, to assess specificity and to confirm that LAMP correctly amplified the target, RT-PCR and PCR was performed (as described above) with the F3 and B3 primers designed for LAMP (see 2.5 below) of SPFMV, SPCSV and SPLCV serving as the forward and reverse primers, respectively.

### 2.4 Quantitative reverse transcription PCR (RT-qPCR) and quantitative PCR (qPCR) assays for RNA and DNA viruses

Reverse transcription Quantitative PCR assays (RT-qPCR) for RNA viruses were performed as described by (Cuellar et al., 2015). The assay was only run to validate and compare results from parallel extraction methods - commercial kit and crude APEG extraction (Section 2.5). TaqMan One Step PCR Master Mix Reagents kit (Applied Biosystems) was used. Briefly, 25 μl reaction volume mixtures with 2 μl of template RNA, 0.4μM each of forward and reverse primer, 0.2μM TaqMan Tamra probe, 12.5 μl of the 2× Master Mix (Applied Biosystems), MMLV (2U/ul) and 10.45 μl nuclease free water (NFW). The following real-time PCR thermal cycling conditions were used: 42 °C for 42 min (cDNA synthesis) and 95 °C for 10 min (hot start activation), followed by 40 cycles of denaturation at 95 °C for 30 s and annealing/extension at 55 °C for 1 min.

Quantitative PCR assays (qPCR) for the DNA virus SPLCV was done as described by Ling et al. (2010). A PCR Master Mix Reagents kit (Applied Biosystems) was used. Briefly, 1 μl of template DNA, 0.4 μM each of forward and reverse primer, 0.2 μM TaqMan Tamra probe, 12.5 μl of the 2× Master Mix (Applied Biosystems), and 10.15 μl nuclease free water were mixed in a 25 μl reaction volume. The following real-time PCR thermal cycler conditions were used: 95 °C for 10 min, followed by 40 cycles of denaturation at 95 °C for 30 s, annealing at 55 °C for 1 min and extension at 72 °C for 1 min. To account for pipetting differences, each sample was run in triplicate on each plate and their threshold cycle (Ct) values averaged during data analysis. In addition, non-template water controls (NTC) as well as positive (total RNA or DNA from virus infected tissue) were included. Real-time PCR reactions were performed on Quant-5 studio (Applied Biosystems).

### 2.5 LAMP primer design and assay optimization

LAMP Designer software – OptiGene (OptiGene Ltd, Horsham, West Sussex, UK) was used to design the primers. LAMP primer parameters were followed as described by (Notomi et al., 2000). To design primers, the coat protein sequences of SPLCV/Sweepoviruses, SPCSV and SPFMV belonging to different lineages around the world were retrieved from GenBank and multiple sequence alignments performed using MEGA 6.0 and Clustal Omega software (http://www.ebi.ac.uk/Tools/msa/clustalo/) to obtain the consensus sequences. Due to diversity of Sweepoviruses, LAMP primers were modified by incorporating degenerate bases wherever necessary. Primers (two outer: F3 and B3; and two inner: FIP and BIP and two F Loop and B Loop) that recognize six distinct regions of the viral coat protein were designed. The sequences of the primer sets are given in Table 2. Primers were synthesized from Invitrogen ™, Macrogene, Eurofins and Inqaba Biotech on different occasions. F1P and B1P, were HPLC purified while F3 and B3 and F Loop and B Loop were desalted.

**Table 2:** Primers tested for loop-mediated isothermal amplification of cytochrome oxidase (COX), *Sweet potato feathery mottle virus* (SPFMV), *Sweet potato cholorotic stunt virus* (SPCSV) and *Sweet potato leaf curl virus* (SPLCV) Sweepoviruses.

Name	Primer	Virus/ Genetic region
SPFMV F3	TACAACGTAAM*CTTGACTGATATGAGT	Coat protein
SPFMV B3	GTTATGTATATTTCTAGTAACRTCAGT	
H SPFMV FIPv2	TGC RGCTGCYTTCATCTGYAWWTGTGGATATGCATTTGATTTYTAYGAGCT	
H SPFMV BIP	AAGAATGCGMRWAATCGGTTGTTTGGGCCTCTCCGTATCYTCTTCTT	
SPFMV F-loop	TTCTTTAGCACGTGYAGGKG	
SPFMV B-loop	TGGAYGGAAACGTCTCCAC	
SPCSV F3	CATCTGAGCAACTGGCTCTT	RNA1 RdRp gene
SPCSV B3	ACCATGAACACATTCTCGAGAT	
H SPCSV FIP	CCTGTAATTTGCCTCACAAAACTCTCCATTCTAACTCACCAGACATTATGTCT	
H SPCSV BIP	GAGATTTTTGCAAGTTTCTACGCATCTCATTCGACGCGTTCTTTTCC	
SPCSV F-loop	GTCTCTTGAATTCATCTTCTTGAC	
SPCSV B-loop	CAAGCTTGGGCAAACCAAAG	
SPCSV_F3_A^a^	CCGATTATGATGGTTCCGATT	RNA1 RdRp gene
SPCSV_B3_A^a^	CGGCGAAAGTCTTCCTAC	
H SPCSV_FIP_A^a^	TGACATACGATGCGACAGCCGGAAGTCGTCATAGATTGGATT	
H SPCSV_BIP_A^a^	CGCGTATGCTGACAGATCTCTTATTATGAGCGCGAAGCAA	
SPCSV_LF_A^a^	CACCTGAAGTACAAATGCTGTG	
SPCSV_LB_A^a^	ATGCTGATGCTGAATCTCTGT	
Sweepo_F_F3	TTGCCAGTCCTTCTGGGC	Coat protein
Sweepo_F_B3	GTAATTTAGATAGGATWTTTTCWCC	
Sweepo_F_FIP	GAAGGCCCAAGYAGAATAGGCAATTTAGGTATTGGGGGTTGACGT	
Sweepo_F_BIP	ATCCATSACATTYTCAGRGCCCTCCTTCTGTITATTCTTCICCTT	
Sweepo_F_LF	TACAGCAACAGTGCTTGGTAT	
Sweepo_F_LB	ARTCRCTGATAATGTCAGGWAC	
COX F3	TATGGGAGCCGTTTTTGC	Cytochrome c oxidase
COX B3	AACTGCTAAGRGCATTCC	
COX FIP	ATGGATTTGRCCTAAAGTTTCAGGGCAGGATTTCACTATTGGGT	
COX BIP	TGCATTTCTTAGGGCTTTCGGATCCRGCGTAAGCATCTG	
COX F-loop	ATGTCCGACCAAAGATTTTACC	
COX B-loop	GTATGCCACGTCGCATTCC	

* Some primers have locations of potential/selected degeneracies or inosine substitutions.

a Improved redesigned SPCSV.

A full factorial design experiment was adopted to expedite the optimization process as multiple input factors were manipulated in determining their effect on a desired output. A series of reactions were performed with the SPFMV, SPCSV and SPLCV primers to obtain optimal conditions. Key considerations in the development and optimization of LAMP assays were: effect of extraction method (Ambion kit versus APEG quick extraction), type of tissue to be analyzed (leaf, root), purity of FIP and BIP (HPLC purified and desalted), specificity of primers, optimal LAMP reaction temperature (evaluated at 61 °C, 63 °C, 65 °C, 67 °C, 69 °C) and LAMP reagents (individual component, ‘wet’, against lyophilized; see Section 2.6).

To assess the effect of extraction method on LAMP assay sensitivity, a serial dilution of 10^−1^ – 10^-8^ was prepared using nuclease free water for the Ambion kit extracted nucleic acid and APEG extract. Serially diluted SPCSV, SPFMV and SPLCV positive samples (CIP_2_L, CIP_3_L, CIP_6_L, CIP_7_L, CIP_8_L, CIP_10_L, CIP_37_K, CIP_43_K, CIP_42_K, CIP_16_K, CIP_28_K and CIP_97_K) were performed. All LAMP assays were run on a rechargeable, portable Genie® II (OptiGene Ltd., UK), at 65 °C for 30 min. A positive reaction was signified by an exponential increase in fluorescence (δR). Peak fluorescence ratio on the amplification rate curve, with a threshold value of 0.02 indicated the time to positivity (T_p)._ Specificity of sweetpotato virus amplicons was determined after a melt curve (T_m_) analysis. This was achieved by heating RT‐LAMP products to 98 °C for 1 min, then cooling to 80 °C and decreasing at 0.05 °C/s. Melting curve analysis was used to distinguish between true and false positive reactions. Similarly, RT-qPCR was conducted for SPCSV and SPFMV, while qPCR for SPLCV was performed as described in Section 2.4.

### 2.6 LAMP ‘wet’ and lyophilized ‘dry’ reagents

The 25 μl ‘wet’ LAMP reaction mix comprised of 15 μl of isothermal master mix ISO-DR002 (Optigene Ltd., UK), 5 μl of 10X the primer set to be tested (SPFMV, SPCSV, SPLCV, final concentration 2X as recommended by Optigene), 4 μl NFW and 1 μl of RNA/DNA template. The 10X primer combination contained 2.0 μM each of forward and reverse inner primers (FIP and BIP respectively), 0.5 μM each of forward and reverse outer primers (F3 and B3, respectively), and 1.0 μM each of forward and reverse loop primers (LF and LB, respectively). The isothermal master mix ISO-DR002-RT (Optigene Ltd., UK) contains a proprietary fast novel DNA polymerase, proprietary thermostable inorganic pyrophosphatase, optimized reaction buffer, MgSO_4,_ dNTPs, and a ds-DNA binding dye (FAM detection channel). On the other hand, for ‘dry’ LAMP the freeze‐dried isothermal master mix ISO-DR004-RT (Optigene Ltd., UK) was used and is similar to ISO-DR002-RT in its final composition but contains undisclosed proprietary reagents. Each reaction was resuspended to 25 μl and LAMP reaction mix comprised 15 μl of resuspension solution, 5 μl of 10x concentrated primer mix, corresponding to the virus to be tested (SPFMV, SPCSV, SPLCV), 4 μl NFW and 1 μl of RNA/DNA template added into individual reaction mix. The ‘wet’ and ‘dry’ assay were both used for lab validation. Lyophilized ‘dry’ LAMP assay was used for on-site detection of SPFMV, SPCSV and SPLCV. LAMP assays were run as described in 2.5.

### 2.7 On-site testing of LAMP assays

Operational performance of ‘dry’ LAMP assays for the detection of SPFMV, SPCSV and SPLCV, respectively, were evaluated on-site in four different geographical conditions in Kenya. These were in the diverse Central, Kakamega, Eastern and Coast regions. Operating temperature average daily max °C (range of daily max) at the time of on-site detection and altitude are given in Table 4. Detection of sweetpotato viruses was evaluated by performing the test on APEG total nucleic acid extracted leaf samples as described above. A total of 24 samples per site were subjected to LAMP assay to test for the three viruses using the lyophilized reagents. Samples were selected randomly and included both symptomatic and asymptomatic plants. The field result for LAMP assay was later compared with RT-PCR/PCR results in the laboratory from the remaining part of the same leaf tested by LAMP in the field, which was preserved with silica-gel for shipment to the lab as described in 2.1 above.

### 2.8 Calculation of diagnostic parameters and agreement between techniques

The *t*-test procedure in SAS (ver. 9.1; SAS Institute Inc., Cary, NC) was performed to compare the differences of extraction methods on LAMP assay results. Diagnostics sensitivity and specificity of LAMP assays were calculated. Sensitivity is the proportion of true positives that are correctly identified by a test, whereas the specificity is the proportion of true negatives that are correctly identified by the test. Sensitivity (SE), specificity (SP) and accuracy (AC), positive and negative predictive values (PPV, NPV) and the kappa index of concordance (k) were calculated as previously described by (Azinheiro et al., 2017). Calculation of diagnostic parameters and agreement between techniques was assessed Cohen’s kappa index (Viera and Garrett, 2005) and McNe-mar’s test, which indicates the proportion of agreement beyond that expected by chance was used to evaluate agreement between techniques. Cohen’s kappa index was categorized as described by (Landis and Koch, 1977); where <0.00 is poor agreement, 0−0.2 is slight agreement, 0.21−0.40 is fair agreement, 0.41−0.60 is moderate agreement, 0.61−0.80 is substantial agreement, and 0.81–1.00 is almost perfect agreement.

## 3 Results

### 3.1 LAMP assay optimization

**Fig. 1:**
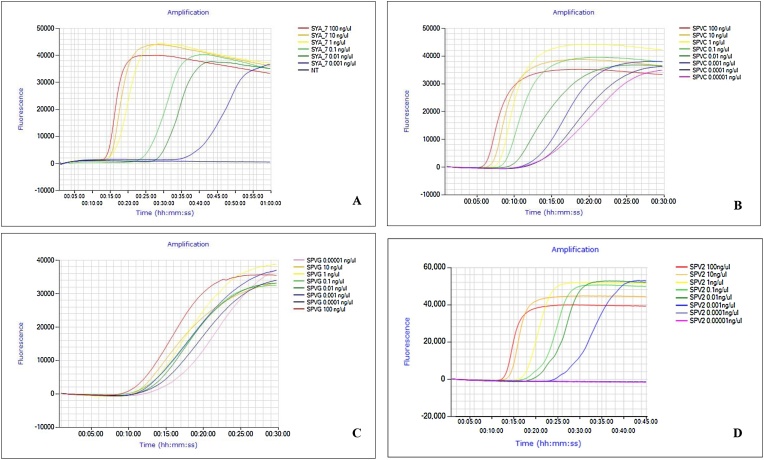
Specificity test with SPFMV primer. The set was tested on four potyviruses. A – Sweet potato feathery mottle virus (SPFMV); B – Sweet potato virus C (SPVC); C – Sweet potato virus G (SPVG) and D – Sweet potato virus 2 (SPV2). Reactions were conducted using kit extracted RNA and diluted in series ranging from 100 ng to 0.00001 ng. Amplification plots displayed an increased TTP with a decrease in RNA concentration.

Initial optimization was done for detection of SPFMV, SPCSV and SPLCV using the reference virus isolates (Table 1). The two primers, F1P and B1P are critical and need to be HPLC purified for assays to function well (data not shown). The optimal temperature for isothermal amplification of all LAMP systems was found to be 65 °C (results not shown). Sample extraction took approximately 3−5 min for APEG crude extraction compared to the Ambion kit extraction time of 30 min.−2 hours. The average time to positivity using the dry LAMP protocol for extraction using the Ambion extraction kit, or APEG protocol (15.3 ± 3.1) were not significantly different between the two extraction methods (17.2 ± 3.7 & 15.3 ± 3.1 respectively in the case of SPFMV; T test: **p** >0.4) (Supplementary Fig. 1). Specificity of the SPFMV primers was evaluated against a panel of other potyviruses; Sweet potato virus G (SPVG), Sweet potato virus C (SPVC), Sweet potato virus 2 (SPV2) (Table 1). The SPFMV primers used in this study were not specific as they amplified all the other potyviruses (Fig. 1). The SPLCV primers amplified all variants of sweetpotato begomoviruses used in our study, whereas the SPCSV primers were specific and only amplified the targeted virus. Amplification of SPFMV was assessed from leaf, stem and root using APEG extraction from greenhouse samples at KEPHIS-Muguga using ‘dry’ LAMP and showed amplification could be achieved from all tissues (Supplementary Fig. 2A). Further, ten samples were tested for viruses in both root and complementing leaf tissue from the same plant in the field at Kiboko and were comparable (Supplementary Fig. 2B an example of SPFMV). LAMP amplified products of the two SPVD components (SPFMV + SPCSV) could clearly be discriminated based on anneal derivative amplification plot showing 86.01 °C ± 0.45 and 83.5 °C ± 0.40 respectively (Supplementary Fig. 3). Results from ‘dry’ and ‘wet’ LAMP were similar except that ‘wet’ LAMP reagents tended to be prone to inhibition in some runs while lyophilized reagents were more reproducible (data not shown).

**Fig. 2:**
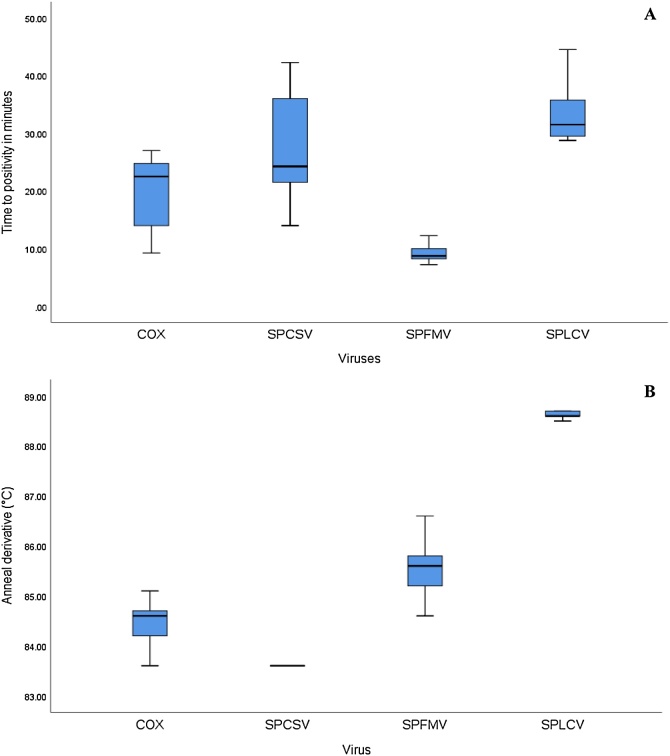
Box-and- whisker plot comparing time to positivity (T_P_) for: Cytochrome oxidase (COX) n = 25, Sweet potato feathery mottle virus (SPFMV) n = 25, Sweet potato cholorotic stunt virus (SPCSV) n = 25 and Sweepoviruses (SPLCV) n = 25. B – Anneal derivative for COX, SPFMV, SPCSV and SPLCV. The bars show the standard deviations in the TTP and anneal derivative respectively. All nucleic acid extractions were done by APEG quick extraction buffer and ran using dry LAMP reagents. Samples are means of the respective positive samples ran over time.

**Fig. 3:**
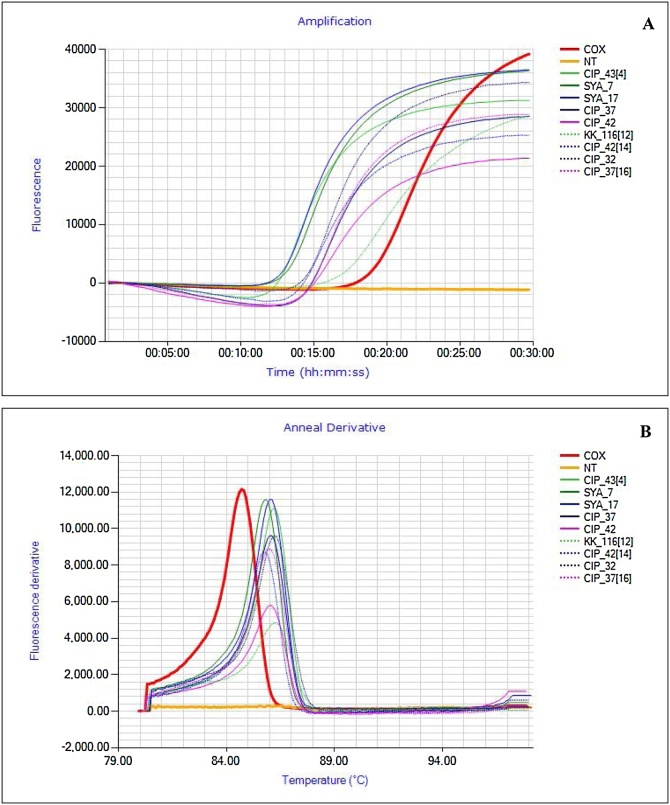
Real time detection of SPFMV positive samples in the field. **A**- The amplification plots showing time positivity (TTP) 13.01 – 17.35 min. **B** – Anneal derivative curves confirming positive reactions of different samples. The products showed a melting temperature of 86.2 °C ± 0.25. The red peak is the positive COX (run in a separate reaction) control and the orange flat line the non-template negative control. All nucleic acid extractions were done by APEG quick extraction buffer.

**Fig. 4:**
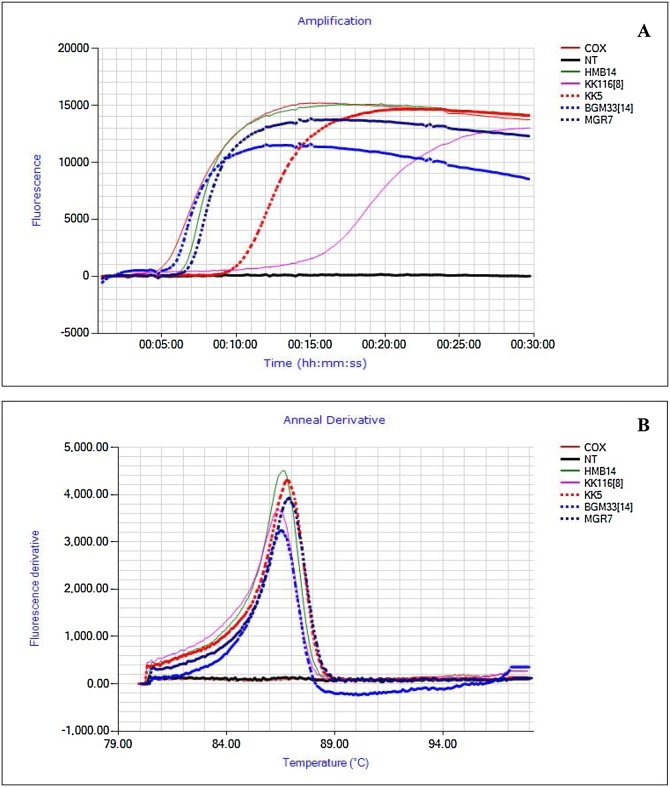
Real time detection of SPLCV for field collected samples. **A**- The amplification curve shows time positivity (TTP) 6.15 – 16.25 min. **B** – Anneal derivative curves confirming positive reactions of different samples. The products showed a melting temperature of 87.6 °C ± 0.35. The red peak is the positive COX control and the orange flat line the non-template negative control. All nucleic acid extractions were done by APEG quick extraction buffer.

**Fig. 5:**
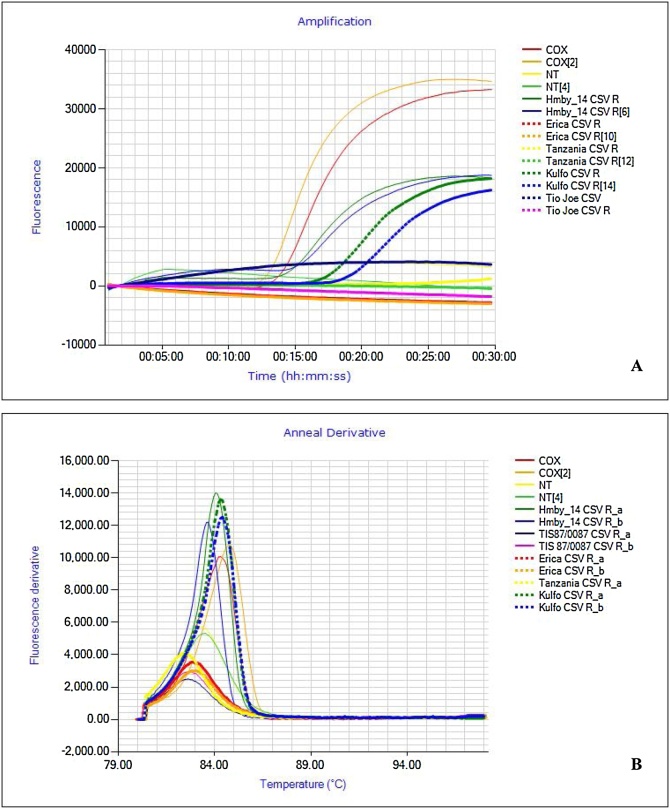
Real time detection of SPCSV) positive samples in the field. **A**- The amplification curve shows time positivity (TTP) 13.20 – 20.45 min. **B** – Anneal derivative curves confirming positive reactions of different samples. The products showed a melting temperature of 84.01 °C ± 0.45. The red peak is the positive COX control and the orange flat line the non-template negative control. All nucleic acid extractions were done by APEG quick extraction buffer.

**Fig. 6:**
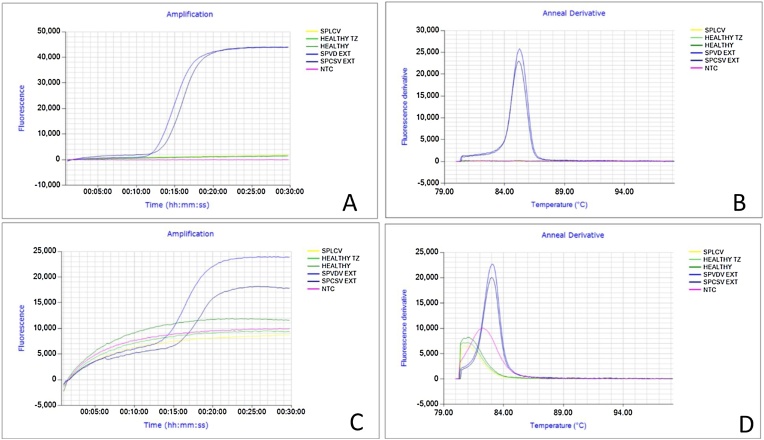
Improved SPCSV primers, with an amplification curve showing a faster time positivity (TTP) of 12 min. and anneal derivative of 83 °C A & B. Contrary, the earlier primer used in this study gave a TTP of 15 min. and an anneal derivative of 85 °C for positive sample but had pseudo anneal derivative curves C & D (Fig. 5) in negative samples.

Results from LAMP assays performed during lab validation and on-site detection in four field sites were in a similar range and displayed following time to positivity (TTP): COX 10–28, SPFMV 5−30 min., SPCSV 15–43 min s and sweepoviruses 28−45 mins (Fig. 2A). To confirm the specificity of the’ dry’ LAMP amplification product, and to distinguish between true and false positive reactions, we performed a melting curve analysis. The mean T_m_ values for specific products for COX, SPFMV, SPCSV, and SPLCV were found to be 84.7 °C ± 0.4, 85.7 °C ± 0.5, 85.6 °C ± 0.45, and 88.7 °C ± 0.4 °C, respectively (Fig. 2B). Examples of ‘dry’ LAMP assay results for SPFMV, SPCSV and SPLCV are shown in (Figs. 3A, 4A and 5 A) and their corresponding T_m_ (Figs. 3B–5B). However, the SPCSV primers resulted in a pseudo anneal derivative in samples that were negative and lacked amplification, although it was at a lower temperature and amplitude than positive samples (Fig. 5B). However, when considering the LAMP amplification curve only, there was 100 % agreement between the ‘dry’ LAMP assays performed and the combined NCM-ELISA/ RT-PCR and PCR results (see 3.2 below). An improved SPCSV primer was designed later in the study that gave a faster TTP and did not have the pseudo anneal derivative (Fig. 6). However, this was not used in the field evaluation.

### 3.2 Analytical sensitivity comparison between ‘dry’ LAMP assay and standard virus indexing

Results from 20 known positive controls and >150 field samples that were earlier tested for the presence of SPFMV, SPCSV and SPLCV by grafting onto *I. setosa* and NCM ELISA and further confirmed by RT-PCR/PCR (see 2.1), were used to select 100 plants to compare with LAMP results. A Fitness-for-Purpose method was adopted, and samples were classified as being positive or negative. Fifty (50) samples were classified as being positive and 50 being negative respectively for (SPFMV, SPCSV and/or SPLCV). The sensitivity, specificity, positive and negative predictive values based on the 100 comparisons were recorded for all the three viruses SPFMV, SPCSV and SPLCV (Table 3). Perfect agreement was evident as Cohen’s kappa value for SPFMV, SPCSV and SPLCV equaled 1 **(**Table 3).

**Table 3:** Diagnostic accuracy parameters assessed (sensitivity, specificity, positive and negative predictive values and kappa analysis) of Loop-mediated isothermal amplification (LAMP) assay for Sweet potato feathery mottle virus (SPFMV), Sweet potato chlorotic stunt virus (SPCSV) and Sweet potato leaf curl virus (SPLCV).

Virus	N	PA	PD	NA	ND	SE	SP	AC	PPV	NPV	k
SPFMV	100	50	0	50	0	100	100	100	100	100	1.00
SPCSV	100	50	0	50	0	100	100	100	100	100	1.00
SPLCV	100	50	0	50	0	100	100	100	100	100	1.00

N - 50 sample were classified as being in positive and 50 being negative.

PA^†^ - positive agreement.

PD^†^ - positive deviation.

NA* - negative agreement.

ND* - negative deviation.

SE* - relative sensitivity (proportion of subjects with the condition who are correctly identified by the test).

SP*- relative specificity (proportion of subjects without the condition who are correctly identified by the test).

AC^†^ - relative accuracy.

PPV* -positive predictive value (proportion of subjects with a positive test result who have the condition).

NPV*- negative predictive value (proportion of subjects with a negative test result who do not have the condition).

k- Kappa index of concordance.

*Proportions as described by Watson and Petrie (2010); the observed frequencies when the gold standard test is compared with an alternative test.

^†^% (at 95 % CI).

^£^All extractions were done by commercial kit extraction method.

### 3.3 On-site detection by LAMP assay and confirmation of results with RT-PCR/PCR

Across all four regions (Muguga, Kakamega, Kiboko and Mtwapa), a total of 96 samples were tested in the field by ‘dry’ LAMP assay for SPFMV, SPCSV and SPLCV and compared to RT-PCR/PCR results of the same leaves in the lab. The results are detailed in Table 4. Two samples from Mtwapa had deteriorated during transportation and hence the difference in number of positive between LAMP and RT-PCR/PCR for SPFMV. Confirmation of field LAMP assays and RT-PCR/PCR for the detection of SPFMV, SPCSV and SPLCV and resultant Cohen’s kappa and Mcnemar's Chi Sq indices for agreement are shown in Table 5. Agreement among methods for SPFMV and SPCSV was very high at 0.9572 and 0.8654, respectively. In contrast, there was also substantial agreement for SPLCV with a Cohen’s kappa index of 0.7368. However, the average overall proportion agreement for SPFMV, SPCSV and SPLCV was 0.9375 (approximately 94 %), with Cohen’s kappa indices of 0.8706 (almost perfect agreement).

**Table 4:** Comparison of the on-site Loop-mediated isothermal amplification (LAMP) assay for in field detection of Sweet potato feathery mottle virus (SPFMV), Sweet potato chlorotic stunt virus (SPCSV) and Sweet potato leaf curl virus (SPLCV); illustrating diagnostic sensitivity (A/(A + C)) and specificity (D/(D + B)) for four geographically dispersed sites in Kenya.

Trial Site		LAMP assay (positive)	LAMP assay (negative)	RT PCR/ PCR (positive)	RT PCR/ PCR (negative)
Muguga	SPFMV	12/24	12/24	12/24	12/24
Kakamega		20/24	4/24	20/24	4/24
Kiboko		16/24	8/24	16/24	8/24
Mtwapa		7/24	17/24	5/24	19/24
Total/Average		55	41	53	43

Muguga	SPCSV	12/24	12/24	12/24	12/24
Kakamega		13/24	11/24	13/24	11/24
Kiboko		5/24	19/24	5/24	19/24
Mtwapa		3/24	21/24	3/24	21/24
Total/Average		33	63	33	63

Muguga	SPLCV	12/24	12/24	12/24	12/24
Kakamega		10/24	14/24	10/24	14/24
Kiboko		2/24	22/24	2/24	22/24
Mtwapa		0/24	24/24	0/24	24/24
Total/Average		24	72	24	72
Operating temperature average daily max °C (range of daily max) at the time of on-site detection and altitude.					

Muguga	Min: 7.6 °C	Max: 23.0 °C	Average: 25.0 °C	1800 masl
Kakamega	Min: 21.6 °C	Max: 32.5 °C	Average: 28.0 °C	1542 masl
Kiboko	Min: 32.6 °C	Max: 41.1 °C	Average: 40.2 °C	956 masl
Mtwapa	Min: 24.0 °C	Max: 36.8 °C	Average: 38.2 °C	22 masl

**Table 5:** Comparison and agreement measures between on-site Loop-mediated isothermal amplification (LAMP) assay and laboratory RT-PCR/PCR for the detection of Sweet potato feathery mottle virus (SPFMV), Sweet potato chlorotic stunt virus (SPCSV) and Sweet potato leaf curl virus (SPLCV).

	Viruses a			
Test comparison statistics	SPFMV	SPCSV	SPLCV	Combined d
Kappa^b^	0.9572 (0.102)	0.8654 (0.1017)	0.7368 (0.1015)	0.8706 (0.0588)
Proportion Positive Agreement	0.9821	0.9143	0.8077	0.9231
Proportion Negative Agreement	0.975	0.9508	0.9286	0.9474
Overall Proportion Agreement^c^	0.9792	0.9375	0.8958	0.9375
Mcnemar’s Chi Sq.	0.5	1.5	0.9	4.5
P (Chi Sq.)	0.4795	0.2207	0.3428	0.0339

a Viruses tested in the field by Loop-mediated isothermal amplification (LAMP) assay and results confirmed by RT_PCR and PCR Sweet potato feathery mottle virus (SPFMV), Sweet potato chlorotic stunt virus (SPCSV) and Sweet potato leaf curl virus (SPLCV).

b Cohen’s kappa index ± standard deviation for kappa = 0 (in brackets).

c Overall proportion agreement diagnostic results by both techniques.

d Combined (SPFMV + SPCSV + SPLCV).

## 4 Discussion

This report constitutes the first validation using diagnostic parameters in the development of field adaptable LAMP assays for SPFMV, SPCSV and SPLCV. Rapid detection is important for disease management in sweetpotato seed systems. LAMP assays were developed in this study for the detection of SPFMV (and related potyviruses), SPCSV and SPLCV (and related begomoviruses), which are the most common viruses infecting sweetpotato in Kenya and sub-saharan Africa, and their operability further evaluated under field conditions in Kenya. Our results show a perfect agreement Cohen’s kappa index of 1 between our LAMP assays for these viruses and the gold standard of grafting to *I. setosa* combined with ELISA and RT-PCR/PCR under laboratory conditions. Njiru (2012) highlighted areas that limited the use of LAMP assays under field conditions. These included: template preparation, lyophilizing LAMP reagents, reliable power source and detection of LAMP products. We discuss improvements in the challenges listed above (Njiru, 2012); in relation to the developed LAMP assay for detection of SPFMV, SPCSV and SPLCV.

Key to the adoption of field testing is the availability of a quick extraction method that negates the need of RNA/DNA purification that is difficult to perform under field conditions. APEG quick extraction has previously been used on blood, insect and plant tissue (Blaser et al., 2018). Howson et al. (2017), demonstrated that for the rapid detection of foot-and–mouth disease virus (FMDV) from clinical samples, a 1:5 dilution of epithelium tissue suspension or serum, and a 1:10 dilution of oesophageal–pharyngeal fluid, in nuclease free water; reduced the inhibitory effect observed by the addition of an undiluted sample to the RT‐LAMP. For rapid detection of sweetpotato viruses, a dilution of 1:10 of crude macerate with deionized water was adopted and results were repeatable and reproducible. At such a dilution, TTP was similar to those obtained with 100 ng of kit purified RNA and when further diluted showed a much slower increase in TTP than kit extracted RNA (Supplementary Fig. 1). In addition, APEG macerates were quite stable, with no significant effect on TTP observed after overnight storage at room temperature (data not shown). At the same time APEG extraction was at least 10 times faster than the use of the Ambion kit (see 3.1). The quick APEG extraction method coupled with field detection of sweetpotato will solve challenges associated with sample storage, transportation and need of expensive and experienced personnel in the diagnostics of sweetpotato viruses.

Another aspect that can limit on-site LAMP detection assays are the use of ‘wet’ LAMP reagents as they require utilization of cold storage to protect the integrity and stability of reagents (Njiru, 2012). We utilized thermostable lyophilized reagents in Genie strip tubes and this obviated the need cold chain storage (Njiru, 2012). Many studies have evaluated the use of thermostable lyophilized reagents that overcome the difficulties of using temperature‐sensitive ‘wet’ reagents in molecular assays (Kurosaki et al., 2016; Howson et al., 2017; Armson et al., 2019). The addition of primers to the LAMP reagents and packaging it into kits is recommended to make it user friendly in a field set up. From the current study, results from ‘dry’ LAMP were similar but somewhat less prone to inhibition than ‘wet’ LAMP reagents. Similar findings were reported by (Armson et al., 2019), who demonstrated that when a sample was added either undiluted or diluted 1/2 in nuclease‐free water, the amplification inhibition observed with ‘wet’ reagents was reduced when replaced with lyophilized reagents. Our ‘dry’ LAMP assays worked well at ambient temperatures on-site for the detection of SPFMV, SPCSV and SPLCV. However once hydrated, reactions must be run quickly as reactivity deteriorated quickly with time (data not shown), thus a recommended approach is to first macerate samples and then prepare the LAMP reagents for immediate addition of macerate and running of the reaction.

The LAMP assays we developed detected all the three viruses with a sensitivity of 100 % as compared to standard virus indexing with a set of 100 plants (Table 3) at Muguga research station. The average time to positivity (TTP) was 5–45 minutes (Fig. 2A) considering both lab and field conditions. This is considerably faster compared to conventional methods: grafting/NCM-ELISA 3–6 months, RT-PCR/PCR – 3 h and qRT-PCR/ qPCR – 2 h respectively (excluding DNA/RNA extraction). Nevertheless, the TTP values of the assays varied between plants, most likely reflecting differences in virus titre depending on how recent a plant was infected, cultivar, co-infections and virus genotype; e.g. Cuellar et al. (2015) showed that begomovirus titers could vary significantly between different isolates, could change over time and also demonstrated that SPFMV/SPLCV titers increased when co-infected with SPCSV under controlled conditions. In addition, efficiency of maceration in APEG buffer due to leaf properties as affected by genotypes and environmental conditions, but also the less accurate approach used to cut and macerate samples than would be used in a lab likely contribute to the variation observed. Consistent with this the TTP for the Cox control gene was also highly variable (Fig. 2A) whereas it is expected to be relatively stable under uniform conditions.

Jiang et al. (2018), developed a LAMP assay for the field detection of SPFMV with a TTP of 70 min and utilized cold storage reagents which are not practical for field application. A shorter TTP from our study could be attributed to a combination of factors: utility of loop primers increases reaction speed as reported by (Li and Ling, 2014), HPLC purified F1P and B1P primers also increase efficiency of LAMP assays (Keremane et al., 2015 & data not shown). In addition, the use of proprietary novel reverse transcriptase, GspSSD 2.0 DNA polymerase ensures a faster TTP compared to Bst DNA polymerase.

We focused our validation on testing of leaf tissues, since that would be the most likely testing approach on a continent where vines are the predominant multiplication material. However, while in the field, we also tested ten root samples from individual plants and roots gave a comparatively fast TTP and results reproduced those obtained with leaf tissue of the same plant (Supplementary Fig. 2). Roots are occasionally used as propagation material in in drought-prone areas and are good reservoirs for viruses (Adikini et al., 2019). Pieces of roots have successfully been used to check for the presence/absence of virus/es by grafting onto *I. setosa,* leading to virus symptom expression (Clark, personal communication). Eguez (2017), evaluated the titre of sweet potato virus C and other potyviruses on three types of organs: roots, stems and leaves. The study established that leaves had a greater relative quantification titer than roots, and stems were intermediate and not significantly different from leaves or roots. We demonstrated that LAMP could detect virus from different sweetpotato organs.

Despite that LAMP assays for detection of SPCSV and SPLCV were specific when considering positive amplification curves, some unexpected observations were witnessed for these assays. The SPCSV assay produced a ‘pseudo anneal derivative’ in a melting curve analysis when the reactions were negative for amplification. Although this is not a large issue since any lack of an amplification curve would lead to consider the reaction negative, it could potentially lead to confusion. This was resolved by the design of new primers, which did not produce the pseudo anneal derivative, and additionally led to more rapid amplification (Fig. 6; Table 2). On the other hand, the SPLCV assays did not produce anneal derivative curves for all the samples where a clear amplification curve was observed (data not shown). We considered them true positives since they also tested positive by PCR, but we do not yet have a theory that could explain this observation. While (Jiang et al., 2018) demonstrated high specificity for his SPFMV assay, we recorded nonspecific reactions for our assay as it also detected other potyviruses - Sweet potato virus C (SPVC), Sweet potato virus 2 (SPV2), Sweet potato virus G (SPVG) (Fig. 1). Though SPFMV strains and other potyviruses are phylogenetically different; they are closely related (Kreuze and Fuentes, 2008; Untiveros et al., 2010), which may explain the cross reaction. Comparison of the SPFMV primers designed in this study, which included several degenerate nucleotide positions to compensate for significant sequence variability found between SPFMV isolates (Table 2), by BLAST and alignment to available SPV2, SPVC, SPVG sequences showed significant identities with a maximum 3 mismatches at non-critical positions for the core F3, B3, FIP and BIP primers. More extensive mismatches were detected in the loop primers, which are however not essential for amplification. The other potyviruses are much less frequent than SPFMV, but because any of the sweetpotato potyviruses can synergize with SPCSV (Kreuze and Fuentes, 2008), we considered the detection of all of them through the same assay as an advantageous property. Further laboratory testing could be conducted to differentiate the potyviruses, if it was required. On the other hand, it remains possible that not all variants of these viruses are detected by our assay as we only tested one each in this study. Detection of SPCSV and SPLCV was not affected by the presence of SPFMV, which often masks the presence of other viruses in sweetpotato, especially potyviruses (Valverde et al., 2007).

The operational field performance of our LAMP assays was evaluated in four different geographical sweetpotato growing regions and results compared to RT-PCR and PCR in the lab, using desiccated leftovers of the same leaves tested in the field (Table 4). Resultant Cohen’s kappa and Mcnemar's Chi Sq indices for agreement among methods for SPFMV and SPCSV were almost perfect. In contrast, the agreement was categorized as substantial in SPLCV (Table 5). The few disagreements between LAMP and PCR were more frequently where PCRs were positive and LAMP negative and the reasons are not immediately obvious as LAMP is generally considered to be about an order of magnitude more sensitive than PCR. It is possible that field conditions as described above might have affected the sensitivity of the assays. The occurrence of false negatives and positives is not desirable but inevitable, as no assay is perfect (Lane et al., 2007). Two samples tested positive for LAMP and negative for RT-PCR/PCR and this was attributed to degraded samples that were not well preserved in silica gel (observable as brown tissue and extract cause by oxidation of the sample).

One of the setbacks of the use of molecular methods is the increased costs compared to the traditional methods. We envisage that LAMP will reduce the time and cost. The current molecular test for sweetpotato viruses costs USD 30 per sample per virus at KEPHIS. Virus indexing and testing for 10 sweetpotato viruses at CIP Lima costs USD 120, which translates to USD 12 per sample and virus tested. Based on costing key reagents, we estimate LAMP to cost USD 6 per sample per virus. Comparatively, the LFD kits for detecting *Phytophthora* spp. costs from ∼USD 7 per test and significantly cheaper than laboratory testing (Lane et al., 2007). Wang and Turechek (2016), optimized recipe for the real-time LAMP assay that saved ∼70 % in cost compared to the commercial kit used in the LAMP assays for other *Xanthomona* species. Abdurahman (personal communication) developed a LAMP assay recipe for the detection of *Ralstonia* from different samples (soil, water, tuber, stem, leaves) that costs approximately USD 3 per sample. This is evident that LAMP assays can be customized to reduce cost. Esmatabadi et al. (2015) listed several platforms that could be incorporated into LAMP detection system: Lateral flow dipstick, enzyme-linked immunosorbent assay (ELISA), and microfluidic chip using antibody-labeled streptavidin–biotin, fluorescent-labeled probes, giant magnetoresistive (GMR) sensors, probe-functionalized nanoparticles, magnetic nanoclusters (MNCs), and line probe assay (LiPA). Some of these platforms have the potential to increase the sensitivity and reduce the cost per reaction (Pankaew et al., 2019). Furthermore, we recommend the pooling of samples and multiplexing LAMP primers when screening to reduce the cost of LAMP. Initial experiments with sweetpotato samples have shown that pooling up to 5 samples can be performed and that the three assays described in this paper can be multiplexed without losing much sensitivity (data not shown). Virus targets can also be multiplexed with the internal control COX assay by using probes labeled with dyes emitting at two different wave-lengths for different target nucleic acids (Tanner et al., 2012), as the realtime Genie-III machine can detect wavelengths at two different channels.

In conclusion, our LAMP assays have the potential to reliably and accurately detect SPFMV, SPCSV and SPLCV. Further, the assay utilizes simple and relatively inexpensive equipment, which renders it promising for use in resource-poor settings. Indeed, we have been able to adapt a kit with single use plastic 1 mL pipettes and 1 μL inoculation loops to simplify the procedure even further without loss of fidelity. We propose this LAMP assay can be used for: field surveys, monitoring of the phytosanitary status of pre-basic seed production in quarantine, or certification program. This can support the production of pathogen-free plant material entering the seed system. However, key to adoption in sub-Saharan Africa will be adequate access to affordable kits. The simplicity and robustness of these kits will make them suitable for the rapid assessment of sweetpotato viruses by plant health inspectors.

## CRediT authorship contribution statement

**Bramwel W. Wanjala:** Data curation, Formal analysis, Investigation, Methodology, Writing - original draft, Writing - review & editing. **Elijah M. Ateka:** Investigation, Methodology, Validation, Writing - review & editing. **Douglas W. Miano:** Investigation, Methodology, Validation, Writing - review & editing. **Segundo Fuentes:** Validation, Writing - review & editing. **Ana Perez:** Validation. **Jan W. Low:** Conceptualization, Writing - review & editing. **Jan F. Kreuze:** Conceptualization, Investigation, Methodology, Validation, Writing - review & editing.

## Declaration of Competing Interest

The authors declare that they have no known competing financial interests or personal relationships that could have appeared to influence the work reported in this paper.
