# Supplementary material for: Loop-Mediated Isothermal Amplification assays for on-site detection of the main sweetpotato infecting viruses
Source: J Virol Methods. 2021 Dec;298:114301. doi: 10.1016/j.jviromet.2021.114301 (PMC8543070; doi:10.1016/j.jviromet.2021.114301)
Supplement: Supplementary file 1 [file mmc1.docx]

Supplementary figure 1: Effect of alkaline polyethylene glycol (APEG) and commercial kit extraction method on Loop-mediated isothermal amplification (LAMP) assay; SPFMV primer for sample SYA_7 using lyophilized LAMP reagents. * T test Time to positivity means for Ambion kit - 17.2±3.7, APEG- 15.3±3.1; **p**=0.4 (**p** >0.05) There is no statistically significant difference between the two extraction methods). Data shows means computed from 5 point dilution series for 5 consecutive assays, error bars shows standard deviation.


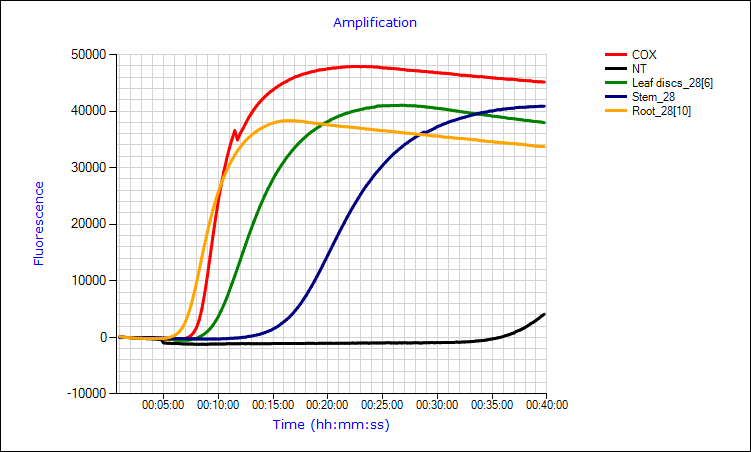

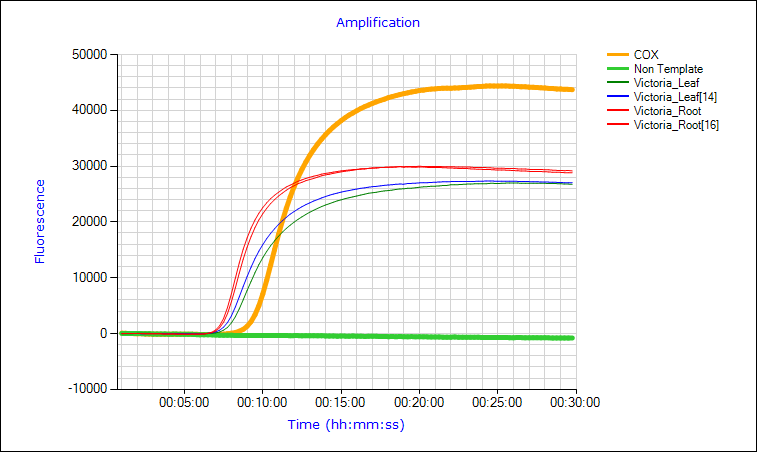


**A**

**B**

Supplementary figure 2. Loop-mediated isothermal amplification (LAMP) assay versatility of SPFMV amplification from leaf, stem and root tissues from the same plant. **A**- The orange curve is the signal of sample extracted from root (TTP 6:30 min). The green curve is a leaf tissue sample extracted from the same plant with a detection time of (TTP 9:10 min) while blue represents stem (TTP 16:20 min). The red curve is the COX control and the black flat line the non-template negative control. **B** – LAMP assay versatility of SPFMV amplification from leaf, and root tissues from the same plant. All nucleic acid extractions were done by APEG quick extraction buffer.


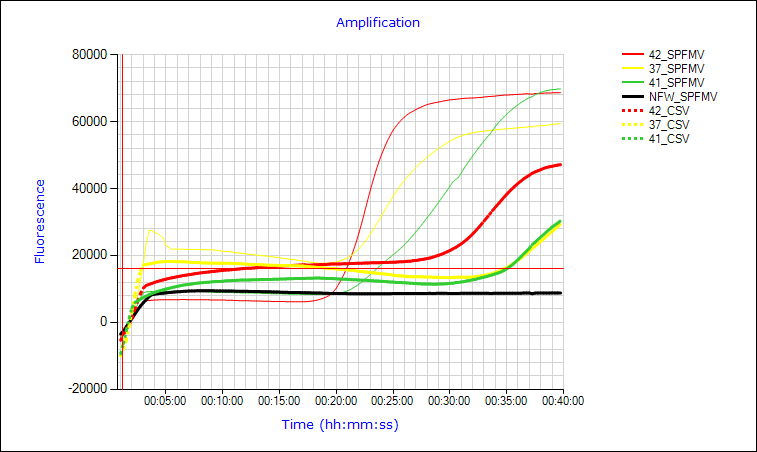

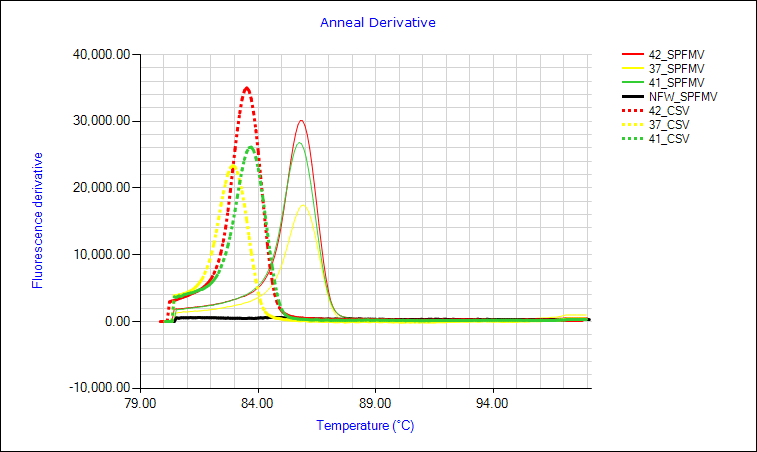


**A**

**B**

Supplementary figure 3: Sweet potato virus disease (SPFMV+SPCSV) positive samples. **A** – Anneal derivative amplification plot for COX, SPFMV and SPCSV (bold) positive samples 42, 37, 41. **B** –Anneal derivative curves confirming positive reactions of SPVD. SPFMV had a melting temperature of 86.01°C ± 0.45, SPCSV (dotted lines) gave 83.5°C ± 0.40 while COX displayed 84.5°C ± 0.40. All nucleic acid extractions were done by APEG quick extraction buffer.
